# The Effects of LIT and MLR-Bf on Immune Biomarkers and Pregnancy Outcomes in Women With Previous Early Recurrent Miscarriage: A Retrospective Study

**DOI:** 10.3389/fimmu.2021.642120

**Published:** 2021-05-04

**Authors:** Lili Meng, Jianping Tan, Tao Du, Xianghua Lin, Shuning Zhang, Xiaolu Nie, Haitian Xie, Jizong Lin, Jianping Zhang, Chen Hui

**Affiliations:** ^1^Department of Obstetrics and Gynecology, Sun Yat-sen Memorial Hospital of Sun Yat-sen University, Guangzhou, China; ^2^Department of Clinical Laboratory, Sun Yat-sen Memorial Hospital of Sun Yat-sen University, Guangzhou, China; ^3^Department of General Surgery, The Third Affiliated Hospital of Sun Yat-sen University, Guangzhou, China

**Keywords:** unexplained recurrent miscarriage, MLB-Rf, rheumatoid biomarkers, successful pregnancy, maternal immunity

## Abstract

**Background:** Immunological failure during pregnancy is considered one of the etiologies of recurrent miscarriage (RM). The decreased production of mixed lymphocyte reaction-blocking factors (MLR-Bf) may play a major role in this condition. Lymphocyte immunotherapy (LIT), which induces the production of MLR-Bf, has been used in treating RM patients since 1984. However, the effectiveness of LIT is currently being heatedly debated. In addition to that, possible changes to the maternal immune system upon induced MLR-Bf production by LIT remains unclear.

**Objectives:** To explore the possible impacts that MLR-Bf may have on the expression of immune biomarkers and pregnancy outcomes, and deduce whether the prevention of miscarriages is possible with LIT or MLR-Bf in RM patients.

**Materials and Methods:** Women with previous early RM (eRM) were enrolled in this retrospective study after they got pregnant again. LIT was implemented before pregnancy and during the first trimester. MLR-Bf and immune biomarkers were checked as the clinical routine. Patients were followed up until 12 gestational weeks. Levels of immune biomarkers and successful pregnancy rates were compared between MLR-Bf^−^ group and MLR-Bf^+^ group stratified by LIT. Independent associations between LIT, or MLR-Bf, and miscarriage were estimated. All data management and analysis were conducted using SPSS 20.0.

**Results:** A total of 1,038 patients, 497 MLR-Bf^−^ (49 cases accepted LIT), and 541 MLR-Bf^+^(463 cases induced by LIT) were included in the study. Percentage of lymphocytes, the ratio of CD4+ T cells/lymphocytes, and levels of some rheumatoid biomarkers (anti-U1-nRNP, anti-SAA-52kd, and anti-CENOP B) were statistically higher in MLR-Bf^+^ group than in MLR-Bf^−^ group among women without LIT. With LIT treatment the successful pregnancy rate was statistically higher in MLR-Bf^+^ group than in MLR-Bf^−^ group (66.7% vs. 51.0%, *P* = 0.028) among women with LIT. Meanwhile, LIT was estimated to have an independent negative association with miscarriage.

**Conclusion:** Upon LIT treament levels of immune biomarkers were different in women with and without MLR-Bf when stratified by whether they received LIT. Not MLR-Bf, but LIT, has an independent protective effect on miscarriage.

## Introduction

Recurrent miscarriage (RM) is one of the reproductive disorders. In China, it is defined as two or more consecutive pregnancy losses before 28 weeks of gestation. Early recurrent miscarriage (eRM) refers to RM that happens within 12 weeks into pregnancy. There are many causes of miscarriage (1–3). Unexplained RM (uRM) is a condition of RM when the etiology of miscarriage is unknown, and it is observed in 50%–75% of RM cases (4). It is broadly accepted that uRM, especially eRM is mainly caused by a defective maternal immune reaction to the fetus (5), with one of the etiologies believed to be decreased production of mixed lymphocyte reaction-blocking factors (MLR-Bf) (6, 7). MLR-Bf is reported as an immunoglobulin G-3 with protective effects for RM patients (8–10). Various mechanisms have been proposed to explain the effectiveness of MLR-Bf in preventing miscarriage. Some of the examples include inhibition of matrix cytotoxic T lymphocytes and blocking cell-mediated immunity(CMI) against fetal antigens (11, 12). Although MLR-Bf can be produced naturally, the prevalence of naturally produced MLR-Bf in Chinese women with RM is <10% (13). Immune lymphocyte therapy (LIT) is an effective treatment for uRM, as it leads to the production of MLR-Bf (14–16). However, its protective effect on women with RM has been controversial in recent years. Even though the effectiveness of LIT on patients experiencing uRM has been reported by several studies (17, 18), the definition for uRM was not standardized, and the sample sizes for these studies were small (19). So far, there is no consistent conclusion for the indications for LIT. Besides, most patients with RM struggles with more than one pathogenic factors (13). The maternal immune system in the maternofetal interface deviates the immune system toward a protective and tolerogenic response (20). Since now, it is not clear whether MLR-Bf affects other aspects of the maternal immune system as a circulative immune protective antibody, or will it guide the maternal body to an even more tolerant status toward the paternal antigen, which may exacerbate underlying autoimmune diseases? For example, the antinuclear antibody(ANA) causes damages to the embryo and leads to adverse pregnancy outcomes, such as miscarriage. This study is therefore designed to elucidate the possible effects of MLR-Bf on the expression of immune biomarkers and pregnancy outcomes and deduce whether LIT or positive MLR-Bf can prevent miscarriages in patients with RM.

## Patients and Methods

### Patients

Women with previous early RM (eRM) were enrolled from June 30, 2018 to June 30, 2020, in this retrospective study after they got pregnant again. Those women were divided into two groups according to whether they had MLR-Bf or not. The inclusion criteria were (1) pregnant with eRM history; (2) gestational age ≤ 12 weeks, and (3) being tested for MLR-Bf. The exclusion criteria were (1) previous experience of sporadic early miscarriage; (2) abnormal embryo chromosomes of the current pregnancy; (3) highest human chorionic gonadotropin (HCG) level of <50 U/L; and (4) lost to follow-up after hospital discharge. A successful pregnancy is defined by one reaching at least 12 weeks of gestation. All patients signed an informed consent form. For patients with eRM history, it is a protocol in our department for all their immune biomarkers to be tested 3 months before pregnancy and once pregnancy is determined. This study was approved by the institutional review board of Sun Yat-Sen Memorial Hospital, Sun Yat-Sen University (No. SYSEC-KY-KS-2021-026).

### Data Classification

#### Maternal Clinical Features

##### Demographic and Maternal Factors

For each pregnancy, the maternal age, weight, and height of the women were recorded. These women were then classified into three groups based on their body mass index (BMI) that was calculated according to their weight and height at the time of hospital admission (<18.5 kg/m^2^, 18.5–24 kg/m^2^, and ≥24 kg/m^2^). Regular menstruation was defined as a regular menstrual cycle that ranges between 21 and 35 days, with the menstruation period lasting from 3 to 7 days, and no dysmenorrhea or irregular menstrual bleeding. Assisted reproduction (including artificial insemination, *in vitro* fertilization, and embryo transfer), multiple pregnancies, and LIT were recorded as yes or no.

##### Reproductive History

The women were categorized into three groups based on previous incidences of early miscarriages: 2, 3, and ≥4; while previous late miscarriage and previous preterm delivery were classified into two categories: 0 and ≥1.

##### Medical History

Detailed medical histories of all patients were obtained, and they were grouped into eight different categories based on past conditions. (1) None: No disease was recorded. Those patients were called women with uRM; (2) Endocrine disorders: polycystic ovary syndrome, diabetes, insulin resistance, thyroid dysfunction, hyperprolactinemia, hyperandrogenemia, adrenal hyperplasia, and other endocrine diseases. (3) Autoimmune disease: systemic lupus erythematosus, scleroderma, rheumatoid arthritis, systemic vasculitis, dermatomyositis, and mixed connective tissue disease. Antiphospholipid syndrome was also classified in this category. (4) Female reproductive tract infections: bacterial vaginosis, mycoplasma infection, chlamydia infection, Candida vaginitis Trichomonas vaginitis, and gonococcal vaginitis. (5) Uterine malformation: congenital uterine dysplasia, uterine fibroids, endometrial adenomyosis, endometriosis, endometrial polyps, and intrauterine adhesions. (6) Chromosomal abnormalities in either spouse (not including polymorphism). (7) Other diseases: diseases associated with other systems or organs not mentioned above. (8) Multiple diseases: More than one disease that was mentioned above.

### Immune Biomarker Measurements

Abbreviations are used to describe the biomarkers, and their reference values and categories are shown in Supplementary Table 1.

MLR-Bf testing was performed with a Sysmex XN9000 and a Lambda Antigen Tray (ELISA). IgA, IgG, IgM, C3, C4, kapp, lamb, IgE, CRP, ASO, RF, ADNaseB, and SAA testings were performed on a SIEMENS BN II automatic protein analyzer using the original matching kit (Nephelometry). White blood cells, lymphocytes, B cells (CD3^−^CD19^+^), NK cells (CD3^−^CD16^+^CD56^+^), CD4^+^ T cells/lymphocytes (CD3^+^CD4^+^), CD8^+^T cells/lymphocytes (CD3^+^CD8^+^), CD3^+^CD4^+^/CD3^+^CD8^+^, and CIK cells (CD3^+^CD56^+^) were tested using the BD FACSCanto II and BD Multitest 6-color TBNK kit (flow cytometry). Anti-U1-nRNP, anti-Sm, anti-SSA-60kd, anti-Ro-52-52kd, anti-SSB, anti-Scl-70, anti-PM-Scl, anti-JO-1, anti-CENOP B, anti-PCNA, ANuA, AHA, anti-RIB-P, and AMA-M2 testings were performed using a AESKU HELIOS and AESKUSLIDES ANA Hep-2 kit (IIFA). Tests for ANA, anti-dsDNA IgG, and Anti_C1q were performed by a EUROBLOT Master and the original matching kit (Euroline).

### Lymphocyte Immunotherapy

The LIT protocol used in this study was published in a previous study (15). Briefly, 50 ml of peripheral blood was collected from partners of the participants by venipuncture directly into heparinized vials. Immediately after blood collection, peripheral mononuclear white blood cells (WBCs) were aseptically separated in the laminar flow by Ficoll–Hypaque gradient centrifugation. WBCs were subsequently washed in saline and resuspended in 1.0 ml of saline solution. Next, 80–100 million WBCs were intradermally injected into the forearm of the pregnant women (1.0 ml divided into five injections, side by side). Injection will be administered every 2–4 weeks, with four injections as a full course of treatment. After that, MLR-Bf was checked to determine the level of production. All the women with eRM who received LIT in this study were treated before pregnancy, and conception will be suggested after one course if MLR-Bf was confirmed positive, or after two courses regardless of MLR-Bf results. Once pregnancy was confirmed, MLR-Bf was checked for the patients, and LIT will be done every 1–2 months up until 12 weeks into pregnancy as a routine in our department.

### Statistical Analysis

Successful pregnancy rates were compared between the MLR-Bf^−^ group and the MLR-Bf^+^ group stratified by clinical features. Levels of immune biomarkers were compared between the MLR-Bf^−^ group and the MLR-Bf^+^ group stratified by LIT. Continuous variables were presented as mean ± standard deviation, or median with range (minimum, maximum). Categorical variables were presented as numbers and percentages. Continuous data were compared by Student's *t*-test, Wilcoxon test, or Mann–Whitney *U*-test, as appropriate, whereas categorical variables were compared using the Chi-square test or Fisher exact test. The predictive contributions of maternal clinical features, immune biomarkers, MLR-Bf, and LIT on miscarriage were analyzed based on an estimation of the crude and the adjusted odds ratios (ORs) from univariate logistic regression and multivariate logistic regression. Variables with a *P* < 0.10 in the univariate regression analysis were considered in the multivariate model using forward stepwise. The possible effect on miscarriage due to interactions between MLR-Bf and LIT was also analyzed and considered in the multivariate logistic regression.

All statistical analyses were performed using the SPSS version 24.0 software (IBM Corp.). Values of *P* < 0.05 were considered to be statistically significant.

## Results

### Cohort Population

**Figure 1:**
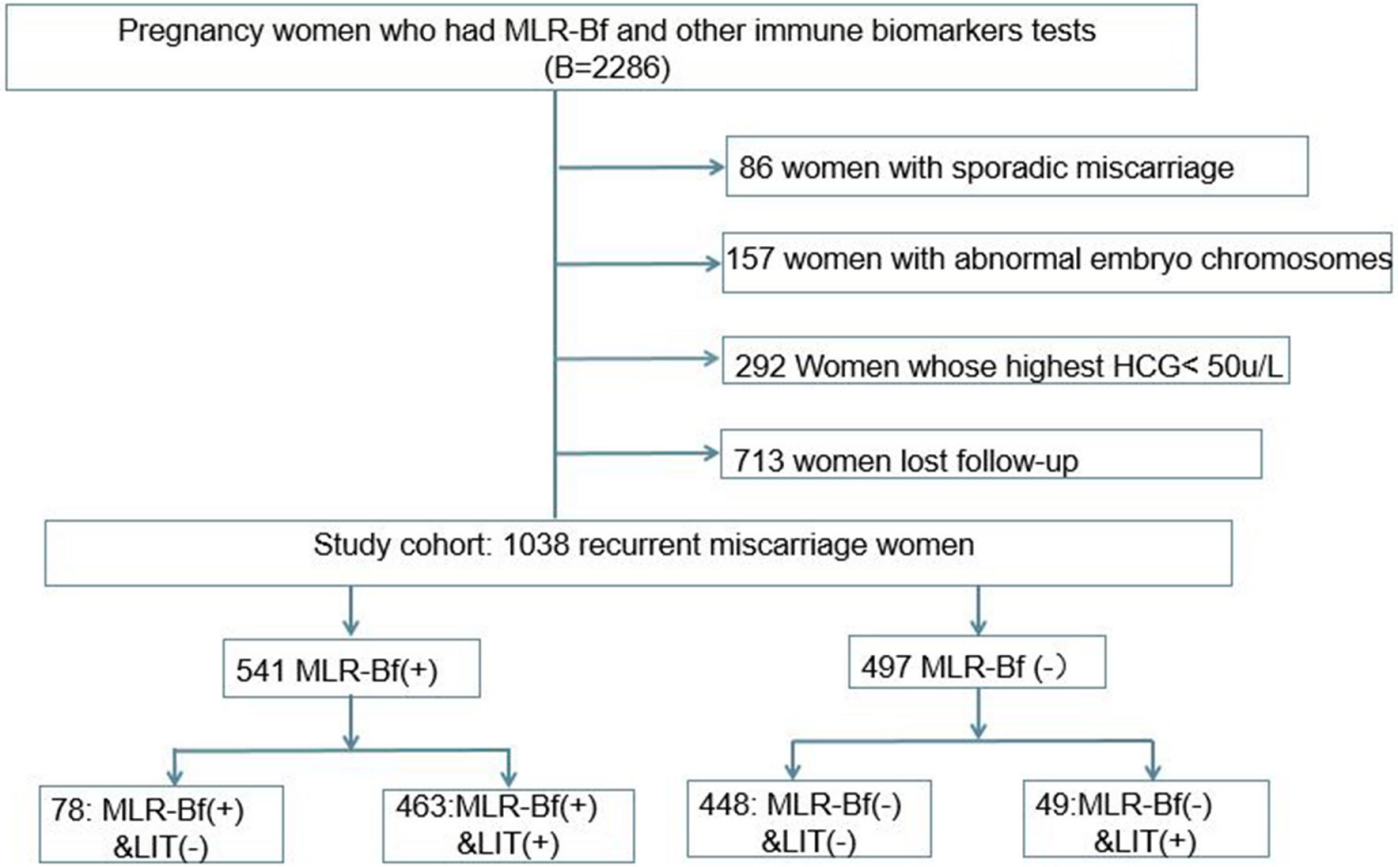
Flowchart of the cohort study.

During the period fixed for this study, a total of 2,286 women with eRM had been admitted to our hospital, and 1,038 of them were included in this retrospective cohort study based on the inclusion and exclusion criteria described (Figure 1). Of the 1,038 patients that were chosen, 497 women were without MLR-Bf, 49 women did not have induced MLR-Bf after LIT, 541 women had MLR-Bf, and 78 women were with naturally produced MLR-Bf.

### Maternal Clinical Features of Participating Patients With and Without Mixed Lymphocyte Reaction-Blocking Factors

The demographic, reproductive history, and medical history of participating patients are shown in Table 1. There were no differences in clinical features between women from MLR-Bf^−^ and MLR-Bf^+^ groups other than age and regularity of menstruation period. The average age of women from MLR-Bf^−^ group was higher than that of MLR-Bf^+^ group, and fewer from MLR-Bf^−^ group had regular menstruation when compared.

**Table 1:** Clinical features of recurrent miscarriage in women with and without MLB-Rf (variables are presented as mean ± SD or number/percentage).

**Variables**	**MLR-Bf ^**−**^ (*N =* 497)**	**MLR-Bf ^**+**^ (*N =* 541)**	***P*-value**
**Age (year)**	**33.2** **±** **5.1**	**32.4** **±** **4.6**	**0.010**
**Height (cm)**	159.1 ± 4.9	159.3 ± 4.9	0.679
**BMI**	22.4 ± 3.9	22.7 ± 3.4	0.164
**Regular menstruation period**			
Yes	**447 (89.9%)**	**505 (93.3%)**	**0.047**
No	**50 (10.1%)**	**36 (6.7%)**	
**Assistant reproduction**			
Yes	119 (23.9%)	104 (19.2%)	0.064
No	378 (76.0%)	437 (80.8%)	
**Multiple pregnancies**			
Yes	23 (4.6%)	26 (4.8%)	0.892
No	464 (95.4%)	515 (95.2%)	
**Medical history**			
No	187 (37.6%)	220 (40.7%)	0.926
Endocrine disorders	70 (14.1%)	63 (11.6%)	
Autoimmune disease	10 (2.0%)	14 (2.6%)	
Infectious disease	8(1.6%)	10 (1.8%)	
Uterine malformation	72(14.5%)	73 (13.5%)	
Abnormal chromosome of the couple	5 (1.0%)	6 (1.1%)	
Other diseases	43(8.7%)	47 (8.7%)	
Multiple diseases	102 (20.5%)	108 (20.1%)	
**Previous early miscarriage**			
2	253 (50.9%)	250 (46.2%)	0.514
3	144 (29.0%)	181 (33.5%)	
≥4	100 (20.1%)	110 (20.3%)	
**Previous late miscarriage**			
0	465 (96.7%)	513 (94.8%)	0.384
≥1	32 (3.3%)	28 (5.2%)	
**Previous preterm birth**			
0	493 (99.2%)	536 (99.1%)	0.836
≥1	4 (0.8%)	5 (0.9%)	
**LIT**			
**Yes**	**49 (9.9%)**	**463 (85.6%)**	**<0.001**
**No**	**488 (90.1)**	**78 (14.4%)**	

### Differences in the Immune Biomarker Levels of Participating Patients With and Without Mixed Lymphocyte Reaction-Blocking Factors: Stratified by Lymphocyte Immunotherapy

The percentage of lymphocytes, the ratio of CD4+ T cells/lymphocytes, and levels of some rheumatoid biomarkers (anti-U1-nRNP, anti-SAA-52kd, and anti-CENOP B) were statistically higher in MLR-Bf^+^ group than in MLR-Bf^−^ group among women without LIT. The only percentage of CD3^+^CD56^+^ cells was statistically lower in patients with MLR-Bf than in without MLR-Bf. No significant statistical differences were identified among the other immune biomarkers when comparisons were made between patients with and without MLR-Bf when stratified by LIT (Table 2).

**Table 2:** Comparison of immune biomarker levels between patients with (*N* = 541) or without (*N* = 497) MLR-Bf (biomarker levels are presented as mean ± SD or medians with lowest and highest values).

	**MLR-Bf^**−**^**	**MLR-Bf^**+**^**	***Z***	***P*-value**
**No LIT**	***N=*** **448**	***N=*** **78 (Naturally produced)**		
IgA (g/L)	2.25 ± 0.77	2.3 ± 0.71	~	0.668
IgG (g/L)	15.6 ± 5.09	16.04 ± 4.71	~	0.546
IgM (g/L)	1.37 ± 0.51	1.38 ± 0.47	~	0.914
C3 (mg/L)	1056.14 ± 183.06	1051.93 ± 180.53	~	0.920
C4 (mg/L)	224.50 ± 74.46	232.27 ± 77.62	~	0.480
kapp (g/L)	3.75 ± 1.16	3.64 ± 1.06	~	0.605
lamb (g/L)	2.01 ± 0.55	2.01 ± 0.53	~	0.960
IgE (IU/mL)	33 (17.3,105.5)	32 (4.56, 686)	−0.290	0.772
C reactive protein (mg/L)	3.13 (3.11, 3.14)	3.11 (3.02, 13.20)	−1.400	0.162
ASO (IU/mL)	9.69 (9.50, 11.30)	9.69 (8.38, 11.30)	−0.122	0.903
RF (IU/mL)	87.8 (54.10, 143.00)	89.35 (49.4, 268.00)	−0.303	0.762
ADNaseB (IU/mL)	93.5 (71.13, 148)	99 (69.6, 247)	−0.509	0.611
SAA (mg/L)	7.05 (4.09, 12.38)	6.8 (2.37, 131)	−0.415	0.678
White blood cells (10^9^/L)	7.14 ± 3.87	7.55 ± 2.09	~	0.365
Lymphocytes (10^9^/L)	**1.90** **±** **0.97**	**2.25** **±** **0.70**	~	**0.003**
T cells (CD3^+^, %)	73.2 ± 6.42	73.78 ± 5.97	~	0.482
B cells (CD3^−^CD19^+^, %)	14.43 ± 4.44	14.71 ± 4.24	~	0.626
NK cells (CD3^−^CD16^+^CD56^+^, %)	12.13 ± 6.44	11.48 ± 5.71	~	0.435
CD4^+^ T cells/lymphocytes (CD3^+^CD4^+^, %)	**41.18** **±** **7.05**	**43.48** **±** **6.49**	~	**0.012**
CD8^+^ T cells/lymphocytes (CD3^+^CD8^+^, %)	26.67 ± 6.39	25.5 ± 5.00	~	0.144
CD3^+^CD4^+^/CD3^+^CD8^+^	1.7 ± 0.63	1.84 ± 0.60	~	0.075
CIK cells (CD3^+^CD56^+^, %)	2.37 ± 2.01	2.33 ± 2.19	~	0.870
Anti-U1-nRNP	**0.00 (0.00, 17.00)**	**1.00 (0.00, 15.00)**	−2.075	**0.038**
Anti-Sm	0.00 (0.00, 5.00)	0.00 (0.00, 9.00)	−0.270	0.787
Anti-SSA-60kd	**0.00 (1.00, 56.00)**	**1.5(0.00, 84.00)**	−2.163	**0.031**
Anti-Ro-52-52kd	0.00 (1.00, 83.00)	2.00 (0.00, 98.00)	−0.945	0.345
Anti-SSB	0.00 (0.00, 40.00)	0.00 (0.00, 4.00)	−0.680	0.497
Anti-Scl-70	0.00 (0.00, 34.00)	1.00 (0.00, 18.00)	−0.723	0.470
Anti-PM-Scl	0.00 (0.00, 9.00)	0.5(0.00, 6.00)	−0.768	0.442
Anti-JO-1	0.00 (0.00, 12.00)	0.00 (0.00, 4.00)	−0.214	0.830
Anti-CENOP B	**0.00 (0.00, 8.00)**	**1.00 (0.00, 105.00)**	−2.040	**0.041**
Anti-PCNA	0.00 (0.00, 11.00)	1.00 (0.00, 10.00)	−1.419	0.156
ANuA	0.00 (0.00, 15.00)	0.00 (0.00, 2.00)	−0.410	0.682
AHA	0.00 (0.00, 41.00)	0.00 (0.00, 55.00)	−0.855	0.392
Anti-RIB-P	0.00 (1.00, 11.00)	1.00 (0.00, 14.00)	−1.483	0.138
AMA-M2	0.00 (1.00, 33.00)	1.00 (0.00, 40.00)	−0.598	0.550
ANA (S/CO value)	1.11 ± 0.74	1.28 ± 1.00	~	0.148
Anti-dsDNA IgG (IU/ml)	0.07 (3.68, 85.12)	4.65 (0.33, 195.5)	−0.373	0.709
Anti_C1q (IU/ml)	0.34 (3.5, 64.89)	3.38 (0.01, 42.23)	−1.162	0.245
**LIT**	***N=*** **49**	***N=*** **463(LIT induced)**		
IgA (g/L)	2.52 ± 0.87	2.26 ± 0.78	~	0.133
IgG (g/L)	17.12 ± 4.54	16.79 ± 5.11	~	0.772
IgM (g/L)	1.46 ± 0.52	1.36 ± 0.51	~	0.360
C3 (mg/L)	1,027.82 ± 139.57	1051.64 ± 177.14	~	0.540
C4 (mg/L)	223.55 ± 57.12	228.77 ± 84.07	~	0.775
kapp (g/L)	3.84 ± 1.10	3.96 ± 1.15	~	0.724
lamb (g/L)	2.04 ± 0.47	2.13 ± 0.60	~	0.602
IgE (IU/ml)	37.50 (4.30, 236)	42.00 (4.00, 4300.00)	−0.250	0.802
C reactive protein (mg/L)	3.13 (3.02, 8.26)	3.13 (3.13, 20.50)	−0.807	0.419
ASO (IU/ml)	9.69 (8.38, 148.00)	9.69 (9.01, 80.90)	−0.169	0.866
RF (IU/ml)	91.50 (49.40, 394.00)	91.65 (43.0, 853)	−0.073	0.942
ADNaseB (IU/ml)	106.00 (69.60, 622.00)	94.00 (50.52, 495)	−0.573	0.570
SAA (mg/L)	7.40 (2.83, 89.10)	6.30 (2.83, 204.00)	−0.777	0.437
White blood cells (10^9^/L)	7.36 ± 3.30	7.54 ± 3.24	~	0.721
Lymphocytes (10^9^/L)	2.07 ± 0.95	2.13 ± 0.87	~	0.635
T cells (CD3^+^, %)	73.09 ± 7.26	72.18 ± 6.78	~	0.431
B cells (CD3^−^CD19^+^, %)	15.55 ± 6.18	14.86 ± 4.39	~	0.372
NK cells (CD3^−^CD16^+^CD56^+^, %)	11.35 ± 5.41	13.24 ± 7.80	~	0.140
CD4^+^ T cells/lymphocytes (CD3^+^CD4^+^, %)	40.66 ± 7.39	40.8 ± 6.69	~	0.905
CD8^+^ T cells/lymphocytes (CD3^+^CD8^+^, %)	25.75 ± 7.22	25.85 ± 5.46	~	0.920
CD3^+^CD4^+^/CD3^+^CD8^+^	1.73 ± 0.63	1.72 ± 0.56	~	0.881
CIK cells (CD3^+^CD56^+^, %)	**3.24** **±** **2.04**	**2.43** **±** **1.90**	~	**0.013**
Anti-U1-nRNP	0.00 (0.00, 4.00)	0.00 (0.00, 33.00)	−0.863	0.388
Anti-Sm	0.50 (0.00, 8.00)	0.00 (0.00, 20.00)	−1.245	0.213
Anti-SSA-60kd	3.00 (0.00, 20.00)	1.00 (0.00, 56.00)	−0.395	0.693
Anti-Ro-52-52kd	7.00 (0.00, 46.00)	3.50(0.00, 98.00)	−0.064	0.949
Anti-SSB	0.00 (0.00, 4.00)	0.00 (0.00, 27.00)	−0.431	0.667
Anti-Scl-70	1.00 (0.00, 2.00)	0.00 (0.00, 14.00)	−0.660	0.509
Anti-PM-Scl	0.00 (0.00, 2.00)	0.00 (0.00, 15.00)	−0.828	0.407
Anti-JO-1	1.00 (0.00, 4.00)	0.00 (0.00, 50.00)	−0.272	0.786
Anti-CENOP B	1.00 (0.00, 2.00)	1.00 (0.00, 44.00)	−0.123	0.902
Anti-PCNA	1.00 (0.00, 3.00)	0.00 (0.00, 15.00)	−1.125	0.260
ANuA	0.00 (0.00, 1.00)	0.00 (0.00, 5.00)	−0.867	0.386
AHA	0.00 (0.00, 14.00)	0.00 (0.00, 45.00)	−0.284	0.776
Anti-RIB-P	1.00 (0.00, 4.00)	0.50 (0.00, 50.00)	−0.025	0.980
AMA-M2	1.50 (0.00, 21.00)	1.00 (0.00, 40.00)	−0.231	0.817
ANA(S/CO value)	1.20 ± 0.68	1.32 ± 0.90	~	0.469
Anti-dsDNA IgG (IU/ml)	4.55 (0.39, 17.51)	3.83 (0.20, 39.51)	−0.119	0.906
Anti_C1q (IU/ml)	3.56 (0.60, 9.29)	3.72 (0.01, 53.77)	−0.137	0.891

### Differences in the Successful Pregnancy Rates of Participating Patients With and Without Mixed Lymphocyte Reaction-Blocking Factors: Stratified by Maternal Clinical Features and Lymphocyte Immunotherapy

As shown in Table 3, the overall rate for successful pregnancy was higher in the MLR-Bf^+^ group (51.7 vs. 65.2%, *P* < 0.001). When stratified by clinical features, it was noticed that for women aged below 35, had a regular period, and undergoing singleton pregnancy, with no medical history, and after LIT, they achieve a higher successful pregnancy rate in MLR-Bf^+^ group than in MLR-Bf^−^ group. In addition to that, higher successful pregnancy rates were also observed in MLR-Bf^+^ group for all BMI categories. Although showing no statistical significance, women in MLR-Bf^+^ group yielded higher successful pregnancy rates in other categories as well.

**Table 3:** Comparison of successful pregnancy rates between patients with and without MLR-Bf stratified by clinical features.

**Variables (%, *n*/*N*)**	**MLR-Bf^**−**^ (*N =* 497)**	**MLR-Bf^**+**^ (*N =* 541)**	***P*-value**
**Advanced age(≥35 years)**			
Yes	40.5 (77/190)	50.3 (83/165)	0.065
No	**58.6 (180/307)**	**71.8 (270/376)**	**<0.001**
**BMI**			
<18.5	**35.3 (18/51)**	**64.6 (31/48)**	**0.008**
18.5– <24	**49.4 (174/352)**	**59.3 (214/361)**	**0.004**
**≥24**	**69.1 (65/94)**	**81.8 (108/132)**	**0.027**
**Regular menstruation period**			
Yes	**51.9 (232/441)**	**65.1 (329/505)**	**<0.001**
No	50.0 (25/50)	66.7 (24/36)	0.124
**Assisted reproduction**			
Yes	60.5 (72/119)	65.4 (68/104)	0.452
No	**48.9 (185/137)**	**65.2 (285/437)**	**<0.001**
**Multiple pregnancies**			
Yes	**78.3 (18/23)**	**80.8 (21/26)**	**0.828**
No	**50.4 (239/474)**	**64.5 (332/515)**	**<0.001**
**Medical history**			
None	**44.4 (83/187)**	**63.6 (140/220)**	**<0.001**
Endocrine disorders	65.7 (46/70)	61.9 (39/63)	0.648
Autoimmune disease	40.0 (4/10)	50.0 (7/14)	0.628
Infectious disease	37.5 (3/8)	70.0 (7/10)	0.168
Abnormal uterine anatomy	45.8 (33/72)	61.6 (45/73)	0.056
Abnormal chromosome of the couple	40.0 (2/5)	83.3 (5/6)	0.137
Other disease	55.8 (24/43)	72.3 (34/47)	0.102
Multiple diseases	60.8 (62/102)	70.4 (76/108)	0.144
**Previous early miscarriage**			
2	**56.9 (144/253)**	**69.2 (173/250)**	**0.011**
3	50.7 (73/144)	60.8 (110/181)	0.119
≥4	**54.0 (54/100)**	**68.2 (75/110)**	**0.019**
**Previous late miscarriage**			
0	**49.7 (231/465)**	**64.9 (333/513)**	**<0.001**
≥1	81.3 (26/32)	71.4 (20/28)	0.370
**Previous preterm birth**			
0	**51.5 (254/493)**	**65.5 (351/536)**	**<0.001**
≥1	75.0 (3/4)	40.0 (2/5)	0.294
**LIT**			
Yes	**51.0 (25/49)**	**66.7 (309/463)**	**0.028**
No	51.8 (232/448)	56.4 (44/78)	0.450
**Total**	**51.7 (253/497)**	**65.2 (353/541)**	**<0.001**

### Variables Related to Miscarriage in Participating Patients: Univariate Logistic Regression Analysis

The crude associations between maternal clinical features/immune biomarkers and miscarriage were presented as percentages/levels or crude ORs in Table 4. Crude ORs for age, weight, BMI, multiple pregnancies, medical history, previous late miscarriage, MLR-Bf, and LIT were statistically significant, while NK cells (*P* = 0.062) and ds-DNA (*P* = 0.097) had a borderline statistical significance.

**Table 4:** Comparison of clinical features, Levels of immune biomarkers, MLR-Bf, and LIT between miscarriage women and successful pregnant women.

**Variables**	**Miscarriage [*N =* 428, %, (*n*/*N*)]**	**Successful pregnancy [*N =* 610, %, (*n*/*N*)]**	***P*-value**	**Crude OR**	**95%CI**	***P*-value**
**Clinical characteristics**						
**Age (year)**	**34.0** **±** **5.3**	**32.0** **±** **3.3**	**<0.001**	**1.094**	**1.065–1.123**	**<0.001**
**Height (cm)**	159.1 ± 4.6	159.3 ± 4.9	0.659	0.986	0.983–1.002	0.421
**BMI**	**21.9** **±** **3.4**	**23.0** **±** **4.1**	**<0.001**	**0.91**	**0.881–0.940**	**<0.001**
**Regular menstruation period (Ref. No)**	8.6 (37/428)	8.0 (49/610)	0.442	0.923	0.591–1.442	0.725
**Assisted reproduction (Ref. No)**	80.6 (345/428)	77.0 (470/610)	0.669	1.238	0.913–1.680	0.170
**Multiple pregnancies (Ref. No)**	**2.3 (10/428)**	**6.4 (39/610)**	**0.002**	**2.885**	**1.409–5.784**	**0.004**
**Medical history (Ref. No)**						
Endocrine disorders	11.2 (48/428)	13.9 (85/610)	0.077	**1.581**	**1.12–2.234**	**0.009**
Autoimmune disease	3.0 (13/428)	1.8 (11/610)		1.082	0.687–1.705	0.733
Infectious disease	1.9 (8/428)	1.6 (10/610)		2.265	0.966–5.31	0.060
Uterine malformation	15.7 (67/428)	12.8 (78/610)		1.533	0.58–4.054	0.389
Abnormal chromosome of the couple	0.9 (4/428)	1.1 (7/610)		**1.646**	**1.067–2.539**	**0.024**
Other disease	7.5 (32/428)	5.8 (58/610)		1.095	0.31–3.865	0.888
Multiple diseases	16.8 (72/428)	22.6 (138/610)		1.057	0.63–1.774	0.832
**Previous early miscarriage (Ref. 2)**						
3	50.1 (127/253)	54.8 (137/250)	0.297	0.918	0.610–1.381	0.681
≥4	34.0 (49/144)	28.7 (52/181)		1.186	0.766–1.838	0.445
**Previous late miscarriage (Ref. No)**	**3.3 (14/428)**	**7.6 (40/610)**	**0.019**	**2.412**	**1.308–2.446**	**0.005**
**Previous preterm birth (Ref. No)**	0.9 (4/428)	0.8 (5/610)	0.996	0.876	0.234–3.281	0.844
**Immune biomarkers**						
IgA (g/L)	2.25 ± 0.81	2.27 ± 0.74	0.705	0.994	0.978–1.239	0.960
IgG (g/L)	16.08 ± 5.27	16.19 ± 4.84	0.798	0.996	0.963–1.030	0.797
IgM (g/L)	1.34 ± 0.50	1.40 ± 0.51	0.134	0.819	0.583–1.148	0.246
C3 (mg/L)	1,043.09 ± 167.33	1,059.64 ± 188.52	0.287	1.000	1.000–1.000	0.615
C4 (mg/L)	224.87 ± 75.43	229.07 ± 81.06	0.536	1.000	0.999–1.001	0.674
kapp (g/L)	3.82 ± 1.16	3.8 ± 1.11	0.836	1.036	0.859–1.250	0.709
Lamb (g/L)	2.06 ± 0.55	2.05 ± 0.57	0.867	1.022	0.701–1.409	0.909
IgE^a^ (IU/ml)	37.00 (4.30, 4,300.00)	34.00 (4.00, 1,130)	0.735	1.137	0.825–1.669	0.374
C reactive protein (mg/L)	3.11 (3.02, 14.80)	3.13 (3.02, 34.8)	0.997	0.954	0.882–1.032	0.240
ASO (IU/ml)	89.35 (49.40, 540.00)	93.05 (49.40, 853.00)	0.522	0.999	0.997–1.001	0.309
RF (IU/mL)	9.69 (8.38, 148.00)	9.69 (8.38, 80.9)	0.794	0.996	0.972–1.021	0.737
ADNaseB (IU/mL)	96.50 (69.60, 1270.00)	96.00 (69.60, 622.00)	0.774	1.001	0.998–1.002	0.798
SAA^a^ (mg/L)	7.40 (2.37, 215.00)	6.35 (2.37, 344.00)	0.025	1.252	0.722–2.171	0.423
White blood cells (10^9^/L)	7.21 ± 3.45	7.45 ± 3.48	0.261	0.978	0.944–1.015	0.238
Lymphocytes (10^9^/L)	2.02 ± 0.94	2.05 ± 0.9	0.669	0.971	0.848–1.111	0.669
T cells (CD3^+^, %)	73.29 ± 6.21	72.41 ± 6.91	0.068	1.020	0.996–1.043	0.101
B cells (CD3^−^CD19^+^, %)	14.57 ± 4.6	14.84 ± 4.70	0.433	0.985	0.953–1.018	0.379
NK cells (CD3^−^CD16^+^CD56^+^, %)	**11.87** **±** **6.25**	**13.01** **±** **7.41**	**0.026**	**0.979**	**0.958–1.001**	**0.062**
CD4^+^ T cells/lymphocytes (CD3^+^CD4^+^, %)	41.42 ± 6.57	40.97 ± 7.01	0.367	1.009	0.988–1.031	0.400
CD8^+^ T cells/lymphocytes (CD3^+^CD8^+^, %)	26.36 ± 6.13	25.94 ± 5.77	0.339	1.008	0.983–1.034	0.517
CD3^+^CD4^+^/CD3^+^CD8^+^	1.71 ± 0.58	1.73 ± 0.59	0.721	0.969	0.754–1.244	0.804
CIK cells (CD3^+^CD56^+^, %)	2.47 ± 2.03	2.42 ± 1.98	0.711	1.001	0.929–1.079	0.977
Anti–U1-nRNP^c^ (Ref. negative)	0.00 (0.00, 28.00)	0.00 (0.00, 33.00)	0.217	2.774	0.582–13.214	0.200
Anti-Sm^b^^,^^c^ (Ref. negative)	0.00 (0.00, 9.00)	0.00 (0.00, 20.00)	0.422	1.000	~	~
Anti-SSA-60kd^c^ (Ref. negative)	1.00 (0.00, 56.00)	1.00 (0.00, 84.00)	0.501	0.933	0.626–1.389	0.733
Anti-Ro-52-52kd^c^ (Ref. negative)	2.00 (0.00, 98.00)	2.00 (0.00, 98.00)	0.932	1.094	0.760–1.575	0.628
Anti-SSB^b^^,^^c^ (Ref. negative)	0.00 (0.00, 40.00)	0.00 (0.00, 4.00)	0.305	~	~	~
Anti-Scl-70^c^ (Ref. negative)	0.50 (0.00, 34.00)	0.00 (0.00, 18.00)	0.296	0.993	0.220–4.484	0.992
Anti-PM-Scl^b^^,^^c^ (Ref. negative)	0.00 (0.00, 9.00)	0.00 (0.00, 15.00)	0.655	~	~	~
Anti-JO-1^c^ (Ref. negative)	0.00 (0.00, 12.00)	0.00 (0.00, 50.00)	0.397	2.247	0.232–21.362	0.485
Anti-CENOP B^c^ (Ref. negative)	1.00 (0.00, 105.00)	1.00 (0.00, 44.00)	0.661	1.877	0.361–9.775	0.454
Anti-PCNA^c^ (Ref. negative)	0.00 (0.00, 11.00)	0.00 (0.00, 15.00)	0.775	2.247	0.232–21.762	0.485
ANuA^b^^,^^c^ (Ref. negative)	0.00 (0.00, 15.00)	0.00 (0.00, 5.00)	0.678	~	~	~
AHA^c^ (Ref. negative)	0.00 (0.00, 55.00)	0.00 (0.00, 41.00)	0.068	0.669	0.289–1.549	0.384
Anti-RIB-P^c^ (Ref. negative)	1.00 (0.00, 50.00)	1.00 (0.00, 20.00)	0.588	1.496	0.271–8.249	0.664
AMA-M2^c^ (Ref. negative)	1.00 (0.00, 40.00)	1.00 (0.00, 40.00)	0.201	0.791	0.488–1.281	0.340
ANA (S/CO value)	1.16 ± 0.81	1.24 ± 0.86	0.303	0.902	0.733–1.111	0.331
Anti-dsDNA IgG (IU/ml)	3.78 (0.07, 52.53)	3.89 (0.20, 195.50)	0.538	0.979	0.954–1.004	**0.097**
Anti_C1q (IU/ml)	3.36 (0.01, 53.77)	3.77(0.60, 64.89)	0.083	1.001	0.972–1.032	0.931
**MLB-Rf (Ref. negative)**	**43.9 (188/428)**	**57.9 (353/610)**	**<0.001**	**0.570**	**0.444–0.732**	**<0.001**
**LIT (Ref. No)**	**41.6 (178/428)**	**54.8 (334/610)**	**<0.001**	**0.588**	**0.458–0.755**	**<0.001**

a *Analysis conducted on the log scale*.

b *Failed to analyze because of small sample size of positive cases*.

C *Levels of immune biomarkers were presented as medians with lowest and highest values, ORs were estimated by categorized variables, and the reference groups were “negative”*.

### Variables Related to Miscarriage in Participating Patients: Multivariate Logistic Regression Analysis

There were interactions between MLR-Bf and LIT on miscarriage. All variables with significant crude predictive contributions (*P* < 0.10) were investigated as independent predictive contributors in multivariate logistic regression models (Table 5). It was discovered that only age, BMI, and LIT contribute as independent predictors of miscarriage. Age as a risk factor had an adjusted OR of 1.079 per year (95% CI: 1.026–1.135). BMI was a protective factor with an adjusted OR of 0.867 (95% CI: 0.816–0.922). LIT was estimated as an independent protective factor, with an adjusted OR of 0.039 (95% CI: 0.243–0.633) (Cox & Snell *R*^2^ = 0.130).

**Table 5:** Risk factors related to miscarriage in RM patients: multivariate analysis.

	**Ref**	**OR**	**95% CI**
**Age (year)**	**~**	**1.079**	**1.026–1.135**
**BMI**	**~**	**0.867**	**0.816–0.922**
**LIT**	**No**	**0.392**	**0.243–0.633**

^*^Interaction of MLR-Bf and LIT was also included in multivariate logistic regression model.

## Discussion

In this study, the possible effects of MLR-Bf on the expression of immune biomarkers and pregnancy outcomes in women with eRM were investigated along with the independent association between LIT, or MLR-Bf, and successful pregnancy. It was discovered that levels of immune biomarkers such as percentage of lymphocytes, the ratio of CD4^+^ T cells/lymphocytes, Anti-SSA-60kd, and anti-CENOP B were higher in women with naturally produced MLR-Bf, and the percentage of CIK (CD3^+^CD56^+^) cells was lower in women with LIT-induced MLR-Bf. Successful pregnancy rates were higher in women with MLR-Bf as well. Besides being higher, successful pregnancy rates were discovered under certain specific maternal clinical features, such as younger age, higher BMI, spontaneous conception, singleton, and in those uRM women and in those who accepted LIT. The percentage of NK(CD3^−^CD16^+^CD56^+^) cells was the only immune biomarker, which found to be different between women who had experienced miscarriage and women with successful pregnancy. However, no independent association was found between NK cells and miscarriage. Age was identified as a risk factor for miscarriage, whereas BMI and LIT were protective factors for the same.

During this study, some rheumatoid immune biomarkers were noticed to be different in patients with and without MLR-Bf when stratified by LIT. This is a field that is rarely investigated. It is well-known that a pregnancy complicated with rheumatologic diseases may influence the fetus and/or neonate (21). Anti-SSA-60kd is one of the biomarkers for Sjögren syndrome. Patients with Sjögren syndrome (82% with anti-SSA) were reported to suffer a higher rate of recurrent miscarriage (30% vs. 3% of controls) (22). Anti-U1-nRNP and Anti-CENOP B are the biomarkers for systemic sclerosis. Nevertheless, we failed to acquire any published article that discusses the relationship between these two antibodies and miscarriage, much less the relationships between them and MLR-Bf. Anti-dsDNA is the biomarker that is used in screening for the flare of systemic lupus erythematosus (SLE). It has been demonstrated throughout the past decades that women with anti-dsDNA face a higher risk of adverse pregnancy outcomes, including miscarriage (23–26). One retrospective study of 100 women with undifferentiated connective tissue disease (UCTD) demonstrated that there was a significant link between disease flare and both anti-dsDNA-positive antibodies at baseline (*P* < 0.01) and disease activity at the beginning of pregnancy (*P* < 0.01) (27). Besides, a subgroup of CD4^+^ T cells (CD4^+^CD25-Foxp^+^) seems to have a significantly positive correlation with SLE activity or anti-dsDNA titer (28). In our study, we found that among women without accepted LIT, both lymphocytes and the ratio of CD4^+^ T cells/lymphocytes were higher in MLR-Bf^+^ group than in MLR-Bf^−^ group. We did not test CD4^+^CD25-Foxp^+^ cells, however, combined with other rheumatoid biomarkers. We advised that more attention should be paid for RM patients with diagnosed SLE or UCTD. It is interesting to find that among women who accepted LIT, CIK (CD3^+^CD56^+^) cells were lower in the LIT-induced MLR-Bf^+^ group than in MLR-Bf^−^ group. CIK (CD3^+^CD56^+^) cells are a subgroup of natural killer T cells (NKT). An abnormally high level of NKT was reported to have associations with RM (29). Marianne found that a decline in the number of elevated blood CD3^+^CD56^+^ NKT cells in response to intravenous immunoglobulin treatment correlates with successful pregnancy (30). Our findings of decreased CIK maybe one of the explanations for the higher successful pregnancy rate in women without MLR-Bf group who received LIT, as no difference was discovered between women with and without MLR-Bf among those who did not receive LIT.

Although the effect of LIT and MLR-Bf remains controversial with the mechanisms yet to be elucidated, the overall successful pregnancy rates were found to be higher in MLR-Bf ^+^ group and in women who had received LIT. In this present study, both MLR-Bf and LIT were included to explore their independent associations with miscarriage. This is something rarely demonstrated before (31–33). With that, LIT was identified as the sole contributor to the differences in successful pregnancy rates. Women with eRM were stratified according to various clinical features to explore the differences in successful pregnancy rates between MLR-Bf^−^ group and MLR-Bf^+^ group. These differences serve as indicators of LIT. Due to the small subgroup sample size, no independent association was made between medical history and miscarriage. However, we found that the successful pregnancy rate of women without medical history, which is considered uRM was higher in MLR-Bf^+^ group than in MLR-Bf^−^ group. This was consistent with other researchers. One study of 140 women with uRM reported that the successful pregnancy rate was 75.8% in women with MLR-Bf, and 30.0% in women without MLR-Bf (*P* < 0.000001) (18). Furthermore, in our study, we found that women with abnormal reproductive tract anatomy who were MLR-Bf^+^ had a higher successful pregnancy rate as well, with a borderline statistical significance (*P* = 0.056). A larger sample size may yield a significant *P*-value. Some immune biomarkers were identified to be differently expressed in MLR-Bf^−^ group and MLR-Bf^+^ group too, but no independent associations were discovered between those biomarkers and miscarriage. To the best of our knowledge, the patients who were involved in this study were all under comprehensive treatments, so it was hypothesized for pregnancy outcomes to be improved.

Non-serological indicators, such as infection factors for their important roles in causing miscarriage and other adverse pregnancy outcomes, were not included in this study (34). Besides, some subsets yielded small sample sizes that reduce the reliability of the statistical analysis. These problems need to be addressed for further studies.

In conclusion, LIT is an effective method to improve pregnancy outcomes for women with eRM. However, patients should be carefully selected. MLR-Bf^+^ is linked to increased levels of some rheumatoid biomarkers, which highlight the necessity for those biomarkers and rheumatic diseases to be screened before and after pregnancy for patients with eRM.

## Data Availability Statement

The original contributions presented in the study are included in the article/Supplementary Material, further inquiries can be directed to the corresponding author/s.

## Ethics Statement

The studies involving human participants were reviewed and approved by Sun Yat-Sen Memorial Hospital. Written informed consent to participate in this study was provided by the participants' legal guardian/next of kin.

## Author Contributions

CH, JL, LM, and JZ conceived and designed the study. LM, JT, and SZ analyzed the data. LM, TD, and JL drafted the first version of the manuscript. XL and TD did the laboratory testing. HX, SZ, and JZ enrolled patients in the study. LM, JT, TD, SZ, XL, JL, JZ, and CH edited the manuscript and read and approved the final version. All authors contributed to the article and approved the submitted version.

## Conflict of Interest

The authors declare that the research was conducted in the absence of any commercial or financial relationships that could be construed as a potential conflict of interest.

## References

[1] Mert UB Murat B Mehmet K Engin Y Necat I Ayhan K. Thrombophilia and recurrent pregnancy loss: the enigma continues. Med Sci Monit. (2018) 24:4288–94. 10.12659/MSM.908832 29932168 PMC6045916

[2] Dong ZR Yan JH Xu FP Yuan JY Jiang H Wang HL. Genome sequencing explores complexity of chromosomal abnormalities in recurrent miscarriage. Am J Hum Genet. (2019) 105:1102–11. 10.1016/j.ajhg.2019.10.003 31679651 PMC6904795

[3] Shafat A Sabhiya M Md NA Shahnaz T Hamed AE Fahad AA. Evaluation of etiology and pregnancy outcome in recurrent miscarriage patients. Saudi J Biol Sci. (2020) 27:2809–17. 10.1016/j.sjbs.2020.06.049 32994741 PMC7499272

[4] Holly BF Danny JS. Recurrent pregnancy loss: etiology, diagnosis, and therapy. Rev Obstet Gynecol. (2009) 2:76–83. 19609401 PMC2709325

[5] Christiansen OB. Reproductive immunology. Mol Immunol. (2013) 55:8–15. 10.1016/j.molimm.2012.08.025 23062611

[6] Kishore R Agarwal S Halder A Das V Shukla BR Agarwal SS. HLA sharing, anti-paternal cytotoxic antibodies and MLR blocking factors in women with recurrent spontaneous abortion. J Obstet Gynaecol Res. (1996) 22:177–83. 10.1111/j.1447-0756.1996.tb00962.x 8697349

[7] Takeuchi S. Immunology of spontaneous abortion and hydatidiform mole. Am J Reprod Immunol. (1980) 1:23–8. 10.1111/j.1600-0897.1980.tb00006.x 7337147

[8] Manoj K Pandey VS Suraksha A. Characterization of mixed lymphocyte reaction blocking antibodies (MLR-Bf) in human pregnancy. BMC Pregnancy Childbirth. (2003) 3:2. 10.1186/1471-2393-3-2 12593676 PMC150574

[9] Manoj K Pandey SA. Induction of MLR-Bf and protection of fetal loss: a current double blind randomized trial of paternal lymphocyte immunization for women with recurrent spontaneous abortion. Clinical Trial Int Immunopharmacol. (2004) 4:289–98. 10.1016/j.intimp.2004.01.001 14996420

[10] Unander A. The role of immunization treatment in preventing recurrent abortion. Transfus Med Rev. (1992) 6:1–16. 10.1016/S0887-7963(92)70151-5 1551001

[11] Shankarkumar U Pradhan VD Patwardhan M Shankarkumar A Ghosh K. Autoantibody profile and other immunological parameters in recurrent spontaneous abortion patients. Niger Med J. (2011) 52:163–6. 10.4103/0300-1652.86126 22082909 PMC3213746

[12] Takakuwa K Kanazawa K Takeuchi S. Production of blocking antibodies by vaccination with husband's lymphocytes in unexplained recurrent aborters: the role in successful pregnancy. Am J Reprod Immunol Microbiol. (1986) 10:1–9. 10.1111/j.1600-0897.1986.tb00001.x 2938488

[13] Meng LL Chen H Tan JP Wang ZH Zhang R Fu S. Evaluation of etiological characteristics of Chinese women with recurrent spontaneous abortions: a single-centre study. Chin Med J. (2011) 124:1310–5. 10.3760/cma.j.issn.0366-6999.2011.09.007 21740739

[14] Liang X Qiu T Qiu LH Wang XP Zhao AM Lin Q. Female third party lymphocytes are effective for immunotherapy of patients with unexplained primary recurrent spontaneous abortion: a retrospective analysis of outcomes. Eur J Contracept Reprod Health care. (2015) 20:428–7. 10.3109/13625187.2015.1046593 25985825

[15] Adachi H Takakuwa K Mitsui T Ishii K Tamura M Tanaka K. Results of immunotherapy for patients with unexplained secondary recurrent abortions. Clin Immunol. (2003) 160:175–80. 10.1016/S1521-6616(02)00044-X 12706403

[16] Beer AE Semprini AE Zhu XY Quebbeman JF. Pregnancy outcome in human couples with recurrent spontaneous abortions: HLA antigen profiles; HLA antigen sharing; female serum MLR blockingfactors; and paternal leukocyte immunization. Exp Clin Immunogenet. (1984) 2:137–53. 2978830

[17] Chernykh N. Mixed lymphocyte reaction blocking factors (MLR-Bf) as potential biomarker for indication and efficacy of paternal lymphocyte immunization in recurrent spontaneous abortion. Arch Gynecol Obstet. (2013) 288:933–37. 10.1007/s00404-013-2832-x 23558562

[18] Taro N Koichi T Izumi O Mami A Tomokazu Y Akira K. Results of immunotherapy for patients with unexplained primary recurrent abortions – prospective non-randomized cohort study. Am J Reprod Immunol. (2007) 58:530–6. 10.1111/j.1600-0897.2007.00536.x 17997752

[19] Hamed H Hamid RN Zeinab L Kobra H Zahra BA Amir F. Lymphocytes immunotherapy for preserving pregnancy: mechanisms and challenges. Am J Reprod Immunol. (2018) 80:e12853. 10.1111/aji.12853 29603821

[20] Leber A Teles A Zenclussen AC. Regulatory T cells and their role in pregnancy. Am J Reprod Immunol. (2010) 63:445–59. 10.1111/j.1600-0897.2010.00821.x 20331584

[21] Iijima S. Fetal and neonatal involvement in maternal rheumatologic disease. J Matern Fetal Neonatal Med. (2018) 31:2079–85. 10.1080/14767058.2017.1334048 28532196

[22] Sara DC Silvia S Angela B Serafina G Cristina G Sergio F. The impact of primary Sjogren's syndrome on pregnancy outcome: our series and review of the literature. Autoimmun Rev. (2013) 13:103–7. 10.1016/j.autrev.2013.09.003 24044939

[23] Ying Y Zhong YP Zhou CQ Xu YW Ding CH Wang Q. A further exploration of the impact of antinuclear antibodies on *in vitro* fertilization-embryo transfer outcome. Am J Reprod Immunol. (2013) 70:221–9. 10.1111/aji.12111 23480310

[24] Morteza M Hadi K Mohammad RA. Prevalence and clinical significance of antinuclear antibodies in Iranian women with unexplained recurrent miscarriage. Iran J Reprod Med. (2014) 12:221–6. 24799884 PMC4009578

[25] Carlo T Federica R Manuela V Adalgisa P Sergio B Francesco R. Antinuclear autoantibodies in women with recurrent pregnancy loss. Am J Reprod Immunol. (2010) 64:384–92. 10.1111/j.1600-0897.2010.00863.x 20482520

[26] Marcelo BC Candice T Manoel S Arlley C Ricardo B. Antinuclear antibodies and recurrent miscarriage: systematic review and meta-analysis. Am J Reprod Immunol. (2020) 83:e13215. 10.1111/aji.13215 31821640

[27] Zucchi D Tani C Monacci F Elefante E Carli L Parma A. Pregnancy and undifferentiated connective tissue disease: outcome and risk of flare in 100 pregnancies. Rheumatology. (2020) 59:1335–9. 10.1093/rheumatology/kez440 31593595

[28] Yin ZJ Ju BM Zhu L Hu N Luo J He M. Increased CD4 + CD25 - Foxp3 + T cells in Chinese systemic lupus erythematosus: correlate with disease activity and organ involvement. Lupus. (2018) 27:2057–68. 10.1177/0961203318804881 30336752

[29] Yuan J Li J Huang SY Sun X. Characterization of the subsets of human NKT-like cells and the expression of Th1/Th2 cytokines in patients with unexplained recurrent spontaneous abortion. J Reprod Immunol. (2015) 110:81–8. 10.1016/j.jri.2015.05.001 26057526

[30] Marianne JVDH Crystal GP Kota H Victor KH David AK. Decline in number of elevated blood CD3(+) CD56(+) NKT cells in response to intravenous immunoglobulin treatment correlates with successful pregnancy. Am J Reprod Immunol. (2007) 58:447–59. 10.1111/j.1600-0897.2007.00529.x 17922698

[31] Manoel S Marcelo BC Marla N Kleber P Ivana L Bianca F. Gestational and perinatal outcomes in recurrent miscarriages couples treated with lymphocyte immunotherapy. Eur J Obstet Gynecol Reprod Biol X. (2019) 7:10036. 10.1016/j.eurox.2019.100036 31403124 PMC6687386

[32] Marcelo BC Fabrício DSC Edward AJ Ricardo B. Risk factors associated with a new pregnancy loss and perinatal outcomes in cases of recurrent miscarriage treated with lymphocyte immunotherapy. J Matern Fetal Neonatal Med. (2015) 28:1082–6. 10.3109/14767058.2014.943175 25005857

[33] Khonina NA Broitman EV Shevela EY Pasman NM Chernykh ER. Mixed lymphocyte reaction blocking factors (MLR-Bf) as potential biomarker for indication and efficacy of paternal lymphocyte immunization in recurrent spontaneous abortion. Arch Gynecol Obstet. (2013) 288:933–7. 23558562 10.1007/s00404-013-2832-x

[34] Janine VDG Dana B Belogolovski A Frishman S Tenenbaum-Gavish K Hadar E. Modulation of cytokine patterns and microbiome during pregnancy in IBD. Gut. (2020) 69:473–86. 10.1136/gutjnl-2019-318263 31167813 PMC7034354

