# Supplementary material for: The Effects of LIT and MLR-Bf on Immune Biomarkers and Pregnancy Outcomes in Women With Previous Early Recurrent Miscarriage: A Retrospective Study
Source: Front Immunol. 2021 May 4;12:642120. doi: 10.3389/fimmu.2021.642120 (PMC8129162; doi:10.3389/fimmu.2021.642120)
Supplement: Supplementary file 1 [file Table_1.DOCX]

**Supplementary table 1. Abbreviations and reference values of immune biomarkers.**

| **Abbreviations** | **Immune biomarkers** | **Normal reference values** |
| --- | --- | --- |
| **IgA(g/L)** | Immunoglobulin A | 0.70-4.00 |
| **IgG(g/L)** | Immunoglobulin G | 7.00-16.00 |
| **IgM(g/L)** | Immunoglobulin M | 0.40-2.30 |
| **C3(mg/L)** | Complement 3 | 650.00-1800.00 |
| **C4(mg/L)** | Complement 4 | 100.00-400.00 |
| **kapp(g/L)** | Free immunoglobulin light chain κ | 1.70-3.70 |
| **lamb(g/L)** | Free immunoglobulin light chain λ | 0.90-2.10 |
| **IgE(IU/mL)** | Immunoglobulin E | 0.00-100.00 |
| **C reactive protein(mg/L)** | ~ | ＜5.00 |
| **ASO(IU/mL)** | Antistreptolysin O | ＜200.00 |
| **RF(IU/mL)** | Rheumatoid factor | ＜20.00 |
| **ADNaseB(IU/mL)** | Anti-streptococcal DNase B | ＜200.00 |
| **SAA (mg/L)** | Serum amyloid | ＜6.80 |
| **White blood cells(10^9^/L)** | ~ | 3.50-9.50 |
| **Lymphocytes(10^9^/L)** | ~ | 1.10-3.20 |
| **Anti-U1-nRNP** | Anti-U1 small ribonucleoprotein antibody | ≤10:negative; 11-24: weak positive; 25-50: Positive; 50: strong positive |
| **Anti-Sm** | Anti-Sm antibody | ≤10:negative; 11-24: weak positive; 25-50: Positive; ≥51: strong positive |
| **Anti-SSA-60kd** | Anti-Sjogren syndrome A antibody (60kd) | ≤10:negative; 11-24: weak positive; 25-50: Positive; ≥52: strong positive |
| **Anti-Ro-52-52kd** | Anti-ribonucleoprotein antibody (52 kd) | ≤10:negative; 11-24: weak positive; 25-50: Positive; ≥53: strong positive |
| **Anti-SSB** | Anti-Sjogren syndrome B antibody | ≤10:negative; 11-24: weak positive; 25-50: Positive; ≥54: strong positive |
| **Anti-Scl-70** | Antibodyanti-scleroderma-70 antibody | ≤10:negative; 11-24: weak positive; 25-50: Positive; ≥55: strong positive |
| **Anti-PM-Scl** | Anti-polymyositis/scleroderma antibody | ≤10:negative; 11-24: weak positive; 25-50: Positive; ≥56: strong positive |
| **Anti-JO-1** | Anti-Jo-1 antibody | ≤10:negative; 11-24: weak positive; 25-50: Positive; ≥57: strong positive |
| **Anti-CENOP B** | Anti-centromeric protein B antibody | ≤10:negative; 11-24: weak positive; 25-50: Positive; ≥58: strong positive |
| **Anti-PCNA** | Anti-PCNA antibody | ≤10:negative; 11-24: weak positive; 25-50: Positive; ≥59: strong positive |
| **ANuA** | Anti-nucleosome antibody | ≤10:negative; 11-24: weak positive; 25-50: Positive; ≥60: strong positive |
| **AHA** | Anti-histone antibody | ≤10:negative; 11-24: weak positive; 25-50: Positive; ≥61: strong positive |
| **Anti-RIB-P** | Anti-ribosomal P protein | ≤10:negative; 11-24: weak positive; 25-50: Positive; ≥62: strong positive |
| **AMA-M2** | Anti-mitochondrial antibody M2 antibody | ≤10:negative; 11-24: weak positive; 25-50: Positive; ≥63: strong positive |
| **ANA (S/CO value)** | Antinuclear antibody | 0.00-1.00 |
| **Anti-dsDNA IgG (IU/mL)** | Anti-double-stranded DNA antibody (IgG) | 0.00-18.00 |
| **Anti_C1q(IU/mL)** | Anti-initiating factor complement 1 antibody | 0.00-10.00 |
|  |  |  |
